# Bisphenol-A Release from Modern Resin-Based Dental Composites: A Time-Dependent In Vitro Assessment

**DOI:** 10.3390/polym18060707

**Published:** 2026-03-14

**Authors:** Angelo Aliberti, Fabiana Di Duca, Mirko Piscopo, Pietro Ausiello, Luigi Ausiello, Alfonso Acerra, Lucia Grumetto

**Affiliations:** 1 Department of Neuroscience, Reproductive Science and Odontostomatological Sciences, University of Naples Federico II, Via Sergio Pansini, 5, 80138 Naples, Italy; ange.aliberti@studenti.unina.it (A.A.); mirk.piscopo@studenti.unina.it (M.P.); 2 Department of Public Health, University of Naples Federico II, Via Sergio Pansini, 5, 80131 Naples, Italy; fabianadiduca91@gmail.com; 3 Department of Pharmacy, School of Medicine and Surgery, University of Naples Federico II, Via D. Montesano, 49, 80131 Naples, Italy; lu.ausiello@studenti.unina.it (L.A.); grumetto@unina.it (L.G.); 4 Department of Medicine, Surgery and Dentistry, University of Salerno, 84084 Salerno, Italy; aacerra@unisa.it; 5 Consorzio Interuniversitario INBB, Viale Medaglie d’Oro, 305, 00136 Rome, Italy

**Keywords:** bisphenol-A, dental materials, artificial saliva, resin-based dental materials, in vitro release, material testing, dental restorations, polymer degradation

## Abstract

Resin-based dental composites are widely used in restorative dentistry; however, concerns persist regarding their potential release of Bisphenol-A (BPA), a compound with recognized endocrine-disrupting activity. This in vitro study evaluated the time-dependent release of BPA from four contemporary resin-based dental filling composites immersed in artificial saliva under different thermal conditions. Disk-shaped specimens (5.5 mm diameter and 2 mm thickness) of *Estelite Sigma Quick*, *Clearfil Majesty ES-2*, *Omnichroma Flow*, and *Luna 2* were incubated in artificial saliva at physiological pH (6.8) at 37 °C and 44 °C. BPA concentrations were quantified after 1, 7, and 28 days using a validated UHPLC–MS/MS method. BPA release was observed for all materials except *Luna 2*, for which it remained below the limit of quantification (LOQ) at all time points and temperatures. Across all BPA-releasing composites, the highest concentrations were observed after 1 day of immersion, particularly at 44 °C. *Estelite Sigma Quick* exhibited the highest BPA release, followed by *Clearfil Majesty ES-2* and *Omnichroma Flow.* BPA release decreased progressively over time for all materials. Statistical analysis confirmed significant effects of material type, temperature, and exposure duration on BPA release (*p* < 0.001). Within the limitations of this in vitro study, BPA release appears to be material-dependent and influenced by thermal conditions and immersion time. Although absolute BPA concentrations were low, these findings highlight the importance of material-specific evaluation and continued monitoring of potential sources of cumulative BPA exposure from restorative dental materials.

## 1. Introduction

Resin-based dental composites are among the most extensively used restorative filling materials in contemporary dentistry due to their favorable esthetic properties, adhesive capability, and compatibility with minimally invasive restorative procedures [1,2]. Continuous advancements in dental resin polymer chemistry and filler technology have improved mechanical performance and clinical longevity of restorations; however, resin-based composites used by dentists to rebuild decayed or fractured teeth remain susceptible over time to physicochemical degradation when exposed to the complex oral environment [3,4,5,6,7]. Polymerization shrinkage, hydrolytic breakdown of the resin matrix, enzymatic activity, mechanical loading and wear contribute to gradual material degradation and may facilitate the release of residual monomers and degradation by-products over time [8,9,10].

In fact, this class of polymer-based restorative materials consists of an organic resin matrix, primarily based on methacrylate monomers, reinforced by inorganic fillers and silane coupling agents that enhance strength and wear resistance [11]. Common monomers include bisphenol-A glycidyl methacrylate (Bis-GMA), ethoxylated bisphenol-A dimethacrylate (Bis-EMA), urethane dimethacrylate (UDMA), and triethylene glycol dimethacrylate (TEGDMA) [12,13]. It should be noted that the potential for bisphenol A (BPA) release is primarily associated with resin systems containing bisphenol-derived monomers such as Bis-GMA or Bis-EMA. In contrast, other commonly used methacrylate monomers, including urethane dimethacrylate (UDMA) and triethylene glycol dimethacrylate (TEGDMA), are not synthesized from BPA and therefore are not considered direct molecular precursors of BPA. Although polymerization aims to achieve a stable cross-linked network, incomplete monomers conversion and long-term polymer exposure to moisture and salivary enzymes may compromise the integrity of the resin matrix, allowing the elution of low-molecular-weight compounds into saliva [14,15,16,17].

Among the substances potentially released from resin-based dental restorative materials, Bisphenol-A (BPA) has attracted scientific and regulatory interest [18]. BPA is a chemical compound widely used in the manufacturing of plastics and epoxy resins, and it is ubiquitously present in the environment commonly found in water dispensers, food packaging and reusable beverage bottles, leading to continuous low-dose exposure in the general population [19,20]. Although BPA is not intentionally added to dental resin composites filling materials, it may be present as a trace impurity derived from the synthesis of bisphenol-based monomers or generated through the degradation of Bis-GMA and Bis-EMA under oral conditions [21,22,23]. The structural relationship between BPA and these commonly used dental monomers provides a mechanistic basis for investigating BPA release from polymer dental restorative materials.

Previous studies have shown that BPA and residual monomers can be detected in saliva soon after placement of resin-based dental restorations, indicating early surface elution [24,25,26]. Although BPA concentrations generally decrease rapidly within the first hours or days, several investigations suggest that prolonged low-level release may occur due to ongoing hydrolytic and enzymatic degradation of the resin matrix [27,28]. Significantly, the extent and duration of BPA release appear to be material-dependent and influenced by multiple factors, including initial BPA impurity content, degree of conversion, curing protocol, resin-monomers composition, and filler characteristics [29,30].

BPA is classified as an endocrine-disrupting chemical (EDC) capable of interacting with estrogen receptors (ERα and ERβ), membrane-associated estrogen receptors, androgen receptors, and thyroid hormone signaling pathways [31,32]. In addition to endocrine activity, BPA has been reported to modulate oxidative stress, inflammatory signaling, mitochondrial function, and epigenetic regulation in experimental models [33,34]. Notably, these effects have been observed at very low concentrations, supporting the concept that chronic, low-dose exposure may be biologically relevant even in the absence of overt toxicity. Considering accumulating toxicological evidence, the European Food Safety Authority (EFSA) has substantially reduced the tolerable daily intake (TDI) for BPA to 0.2 ng/kg/day, underscoring the importance of minimizing all avoidable exposure sources [35]. When the concentrations measured in this study are interpreted within the context of the EFSA tolerable daily intake (TDI), it should be noted that the present results represent in vitro release values rather than direct estimates of systemic exposure. Therefore, the measured BPA concentrations cannot be directly translated into daily intake values. Nevertheless, the relatively low concentrations detected under the tested conditions suggest that BPA release from contemporary resin-based composites is limited and strongly influenced by material composition and environmental conditions. In this context, evaluating potential sources of BPA exposure from commonly used dental restorative materials has become increasingly relevant, particularly given the widespread use of resin-based composites and their direct and prolonged contact with saliva and oral tissues.

Human exposure to BPA is inherently cumulative, arising from multiple dietary, environmental, and consumer-related sources [36,37,38,39,40,41]. Within this broader exposure framework, dental monomers and polymer restorative materials represent a localized but clinically relevant source, as they are in direct contact with saliva and oral tissues and may release BPA in close temporal proximity to placement procedures. Although the contribution of dental composites to total BPA intake is generally considered limited, their potential role in sustained low-level exposure warrants careful evaluation, particularly in the context of increasingly stringent regulatory thresholds and widespread use of resin-based dental restorative materials [42].

To address concerns related to BPA exposure, some dental manufacturers have introduced restorative materials marketed as “BPA-free,” often through the replacement or reduction of Bis-GMA in favor of alternative monomers such as urethane dimethacrylate (UDMA) [43]. Nevertheless, recent evidence suggests that dental filling polymer materials labeled as BPA-free may still release trace amounts of BPA or alternative monomers with biological activity, highlighting the need for independent analytical assessment rather than reliance on compositional claims alone [44,45,46].

In vitro testing in artificial saliva provides a controlled and reproducible approach for evaluating elution behavior of resin-based dental materials under standardized conditions [47,48,49]. In this context, the observation period of up to 28 days represents an extended experimental timeframe beyond the initial release phase within the controlled in vitro conditions of the present study and should not be interpreted as reflecting the clinical service lifetime of dental restorations [27].

This in vitro study aims to evaluate and compare the time-dependent release of BPA from four contemporary resin-based dental composites, *Luna 2* (SDI), *Clearfil Majesty ES-2* (Kuraray Noritake Dental), *Omnichroma Flow* (Tokuyama Dental), and *Estelite Sigma Quick* (Tokuyama Dental), when immersed in artificial saliva under controlled temperature conditions (37 °C and 44 °C). BPA concentrations were quantified at multiple time points (1 day, 7 and 28 days) using liquid chromatography–tandem mass spectrometry (LC–MS/MS), allowing a standardized comparison of BPA release behavior among different modern restorative materials. Based on these considerations, the null hypothesis was that BPA release would not differ among the tested resin-based materials, temperatures (37 °C and 44 °C), or exposure times (1 day, 7 days and 28 days) in artificial saliva.

## 2. Materials and Methods

### 2.1. Specimen Preparation

Four commercially available restorative materials were evaluated. Information on the selected materials and their chemical compositions are reported in Table 1. Specimen fabrication was carried out in accordance with the manufacturers’ recommendations. Samples of *Omnichroma Flow* (Tokuyama), *Estelite Sigma Quick* (Tokuyama), *Clearfil Majesty ES-2* (Kuraray) and *Luna 2* (SDI) were fabricated according to the manufacturer’s instructions. Twelve specimen disks (5.5 mm diameter and 2 mm thickness as reported by De Nys S. et al. [23]) of each material were prepared in custom-made Teflon molds. Based on these dimensions, the calculated surface area of each specimen was approximately 95 mm^2^, with a volume of approximately 47.5 mm^3^, resulting in a surface area-to-volume ratio of approximately 2.0 mm^−1^. Standardized specimen dimensions were used to ensure comparable exposed surface areas among the tested materials. The top and bottom surfaces were covered with a glass plate during light curing, and gentle pressure was applied to displace excess material and obtain flat and standardized specimen surfaces. The use of a glass plate also helps minimize the formation of the oxygen inhibition layer at the specimen surface. Samples were polymerized for 20 s by light-curing using a LED light-curing unit (RADII-CAL CX Collimated Led Curing Light, SDI, Bayswater, Australia) with the light guide positioned in contact with the glass plate covering the specimen surface (0 mm distance). The curing light delivered an average intensity of 1200 mW/cm^2^, as verified using a LED radiometer (SDI, Victoria, Australia). After curing, the disks were removed from the molds and both the top and bottom surfaces were polished with 800-grit abrasive paper using a water-cooled rotating polishing machine (Ecomet 30, Buehler Ltd., Lake Bluff, IL, USA). This procedure was performed to remove the superficial resin layer potentially containing residual unpolymerized monomers associated with the oxygen inhibition layer and to standardize surface conditions prior to the BPA release analysis, as commonly performed in in vitro studies investigating monomer release from resin-based dental materials [22,23,27].

The specimens were equally allocated to two temperature conditions (37 °C and 44 °C), resulting in six specimens per material for each temperature group. Overall, a total of 48 specimens were included in the experimental analysis, consistent with the sample size determined by the a priori power analysis.

### 2.2. Reagents and Chemicals

Bisphenol-A (BPA, purity ≥ 99%) used as standard for method validation was purchased from Sigma-Aldrich (Milan, Italy). Methanol (MeOH, HPLC grade ≥ 99.9%) was also obtained from Sigma-Aldrich (Milan, Italy). Milli-Q water was produced in-house, and its conductivity was 0.055 μS cm^−1^ at 25 °C (resistivity equals 18.2 MΩ·cm). Ultrapure water was produced in the laboratory using Elix Essential Water Purification System (Merck Millipore, Burlington, MA, USA). Stock solution of BPA was prepared by accurately weighing 2.0 ± 0.1 mg of analyte, in dark glass vials, and dissolving them in 5 mL of MeOH. The standard solution was stored at −20 °C for up to 4 months. Working standard solutions of BPA were prepared by combining aliquots of each stock solution and diluting them in MeOH to obtain a final concentration of 0.1, 0.25, 0.5, 1, 2.5, 5.0, 10.0 µg L^−1^. After preparation, the working solutions were stored at −20 °C and, before use, were kept at room temperature and vortexed for 1 min.

### 2.3. Extraction Protocol and Sample Analysis for Bisphenol-A Release

After preparation, each sample was placed in glass vials. Then, 2 mL of a commercially available artificial saliva (SAGF), whose composition is based on the Fusayama Meyer formulation and adjusted to pH 6.8, were added to each specimen [50]. Afterwards, following the procedure outlined in previous studies [51,52] the samples were incubated at 37 °C or 44 °C for defined periods of 1 day, 7 days, and 28 days. Fusayama Meyer’s artificial saliva solution components are indicated in Table 2.

Regarding temperature conditions, existing studies indicate that 37 °C reflects the mean intraoral temperature, whereas 44 °C was selected to reproduce temporary increases associated with the intake of hot beverages or febrile conditions [53,54].

At fixed time intervals (1 day, 7 and 28 days), aliquots of 200 µL were collected and immediately replaced with the same volume of fresh SAGF medium. The collected samples were transferred in glass vials with inserts and analyzed to quantify the amounts of BPA with LC-ESI-QqQ-MS/MS. BPA concentrations were measured in the incubation medium at each time point following partial renewal of the storage solution and therefore represent interval concentrations rather than cumulative release. Given that all specimens were prepared with identical dimensions, BPA concentrations were compared directly across materials.

Since BPA could be released from plastic laboratory instruments, all procedures were performed using glassware and washed only with organic solvents and water, to minimize BPA contamination. Procedural blanks, consisting of artificial saliva incubated in glass vials under identical experimental conditions but without specimens, were analyzed and served as external negative controls to exclude potential background BPA contamination arising from the storage medium, containers, airborne sources, or the analytical workflow.

### 2.4. LC-MS/MS Analysis

Analyses were conducted with an Agilent 6470 LC/ESI-TQ system (Agilent Technologies, Santa Clara, CA, USA) equipped with a Jet Stream ion source operated in negative ion mode. Chromatographic separation was performed with an Agilent 1290 Series UHPLC (Santa Clara, CA, USA), equipped with a Luna Polar 1.7 µm, 100 Å, 50 mm × 2.1 mm stainless steel column (Phenomenex, Torrance, CA, USA). During the analysis, the flow rate was set at 0.400 mL/minute and the column temperature at 45 °C. A sample volume of 5 µL was injected. Separation was achieved by a linear gradient from 0.01% acetic acid in ultrapure water to 0.01% acetic acid in MeOH as displayed in Table 3.

The time for post-run column re-equilibration was fixed at 2 min. The mass spectrometer was periodically calibrated in the mass range 112.99–2833.87 amu. Mass Hunter Workstation software (version B.08.00, Agilent, Santa Clara, CA, USA) was used for data acquisition and processing. Analyses were conducted in multiple-reaction monitoring (MRM) mode. Flow injections of standard solutions of BPA at 1000.0 ng/mL were employed to optimize source parameters with LC flow conditions. The following experimental parameters were optimized: Gas temperature 200 °C, Gas flow: 11 L/min, Nebulizer: 45 psi, Sheath gas temperature 350 °C, Sheat gas flow: 12 L/min, Ion spray voltage −3500 V, Nozzle voltage 2000 V. The MRM transition was optimized by acquiring the product ion spectra and using Optimizer software (version B.08.00, Agilent Technologies, Santa Clara, CA, USA) provided by the LC-MS manufacturer (Agilent, Santa Clara, CA, USA). Tandem mass spectrometry analyses were performed in multiple reaction and negative ionization monitoring mode (-MRM). For precursor ion (mass Q1), two product ions (masses Q3) were selected, one for quantification and the other for confirmation, identifying a quantifier ion (Q) and a qualifier ion (q). Both Q1 and Q3 were operated at unity resolution with a cell accelerator voltage of 7 V, and 150 ms was the dwell time allowed for each transition. Identification of the BPA was based on comparison of the retention time (tR) of the chromatographic peaks of the quantifying and qualifying ions with the peaks of the reference standards. UHPLC-MS/MS Quantifier and Qualifier transitions used for BPA are described in Table 4 below. Data were collected and processed using MassHunter Workstation (Agilent, Santa Clara, CA, USA).

Method sensitivity was assessed by calculating the limit of detection (LOD) and the limit of quantification (LOQ) using signal-to-noise criteria. The LOD was defined as the analyte concentration producing a signal-to-noise ratio of 3, whereas the LOQ was established at a signal-to-noise ratio of 10. Under the adopted experimental conditions, the LOD and LOQ for BPA were approximately 0.03 µg/L and 0.10 µg/L, respectively. These parameters define the lowest BPA concentrations that can be confidently detected and quantified, ensuring the suitability of the analytical method for trace-level BPA analysis. BPA concentrations below the LOQ were reported as <LOQ and were not included in quantitative statistical comparisons, since concentrations below the quantification limit cannot be reliably expressed as precise numerical values. In the case of Luna 2, all measurements remained below the LOQ and were therefore reported descriptively without inclusion in the mixed-effects statistical model. This approach is consistent with common practices in analytical chemistry when all measurements for a specific group fall below the quantification limit, as substitution methods may introduce artificial numerical values that are not directly supported by analytical measurements.

### 2.5. Statistical Analysis

A priori power analysis was performed using G*Power (version 3.1.9.7, Heinrich Heine University Düsseldorf, Düsseldorf, NRW, Germany) to determine the adequacy of the sample size for detecting differences in BPA release among restorative materials and experimental conditions. Considering a mixed-design ANOVA model with four groups (materials), two between-subjects temperature conditions (37 °C and 44 °C), and three repeated time points (1, 7, and 28 days), the analysis was conducted assuming a medium effect size (f = 0.25), an α level of 0.05, and a desired statistical power of 0.80. The selection of a medium effect size (f = 0.25) followed conventional recommendations for ANOVA-based experimental designs as described by Cohen and was considered appropriate given the moderate differences in monomer or BPA release typically reported in previous dental materials studies. Under these parameters, the minimum required sample size was calculated to be N = 48 total specimens (n = 6 per material for each temperature condition), which matches the number of samples included in the present study and is therefore considered adequate to detect statistically significant differences in BPA release across materials, temperatures, and exposure durations.

Statistical analysis was performed using R software (version 4.3.1, R Foundation for Statistical Computing, Vienna, Austria), implementing a mixed-effects modeling approach equivalent to that available in STATA. A mixed-effects model was employed to evaluate the effect of composite material, temperature, and exposure time on BPA release. BPA concentration was modeled as the dependent variable, with material type, temperature, and time included as fixed factors. Specimen identification number was incorporated as a random effect to account for repeated measurements over time. All measurements were conducted at a constant pH of 6.8.

Two-way and three-way interactions among the fixed factors were tested. Model assumptions were verified through residual analysis. The statistical significance of each fixed factor and their interactions was assessed using the Wald test. Post hoc pairwise comparisons were performed when significant main effects or interactions were detected, with adjustment for multiple comparisons using the Bonferroni correction method. Descriptive statistics, including mean, standard error, minimum, and maximum values, were calculated for each composite material.

Normality of the BPA concentration data distribution was evaluated using the Shapiro–Wilk test for each material separately. Statistical significance was set at *p* < 0.05 for all analyses.

## 3. Results

### 3.1. BPA Release

**Figure 1:**
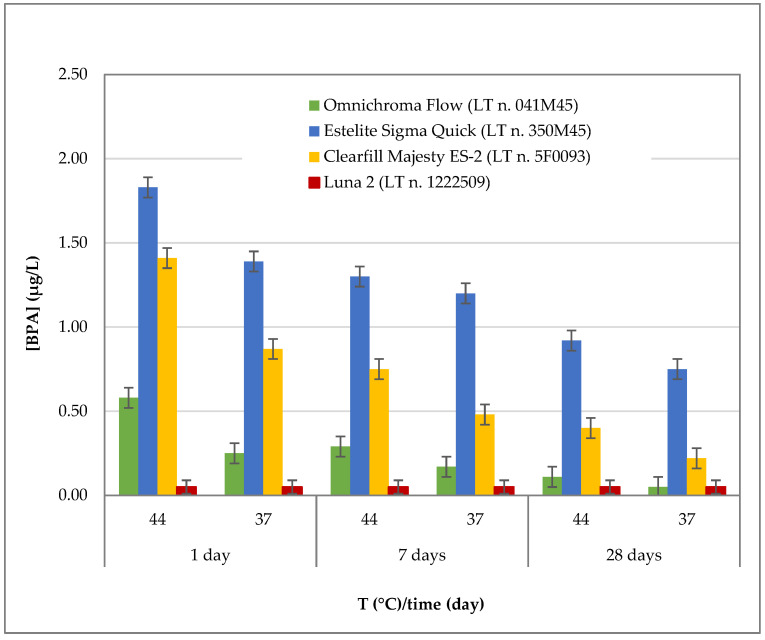
Comparison of BPA release from all materials in SAGF (pH 6.8) at two temperatures (37 °C and 44 °C) and three observation times (1, 7 and 28 days).

**Figure 2:**
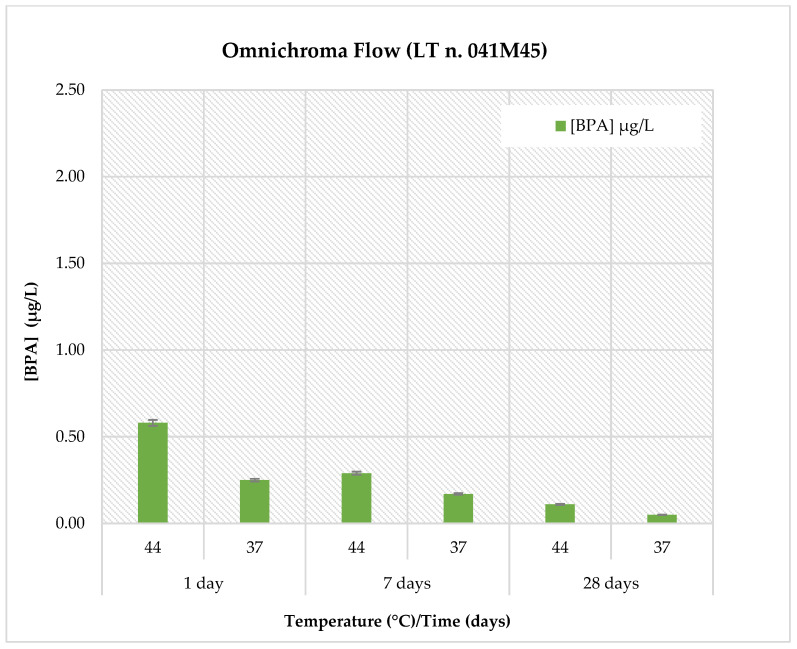
Comparison of BPA release in Omnichroma Flow material at pH = 6.8, two temperatures (44 °C and 37 °C) and at three different observation times (1, 7 and 28 days).

**Figure 3:**
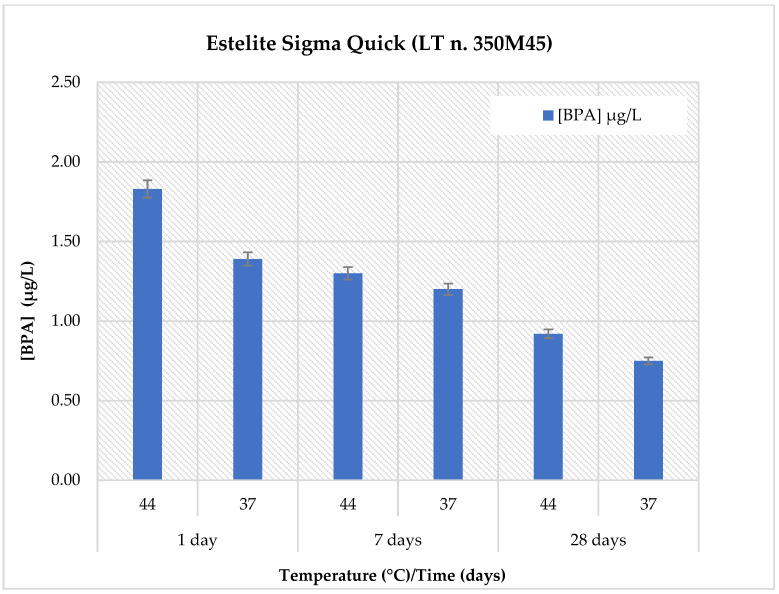
Comparison of BPA release in *Estelite Sigma Quick* material at pH = 6.8, two temperatures (44 °C and 37 °C) and at three different observation times (1, 7 and 28 days).

**Figure 4:**
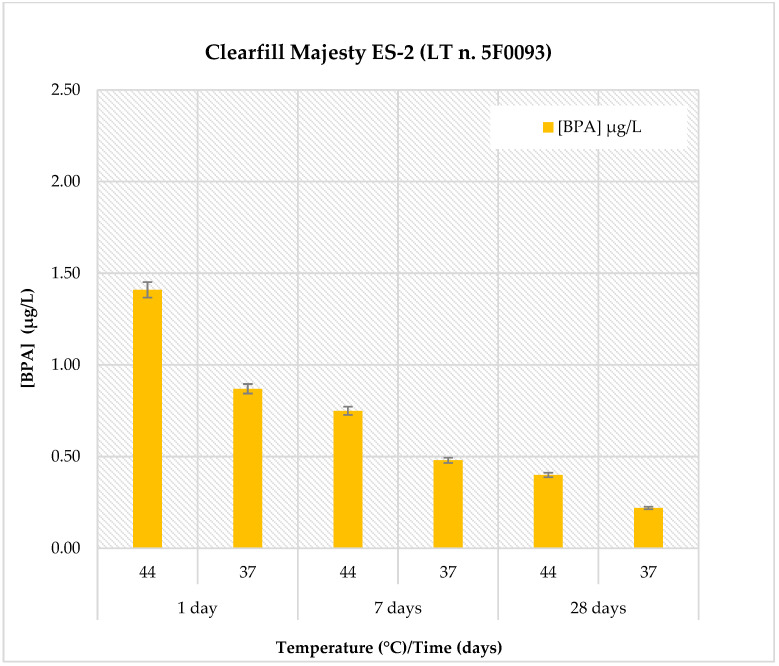
Comparison of BPA release in *Clearfil Majesty ES-2* material at pH = 6.8, two temperatures (44 °C and 37 °C) and at three different observation times (1, 7 and 28 days).

**Figure 5:**
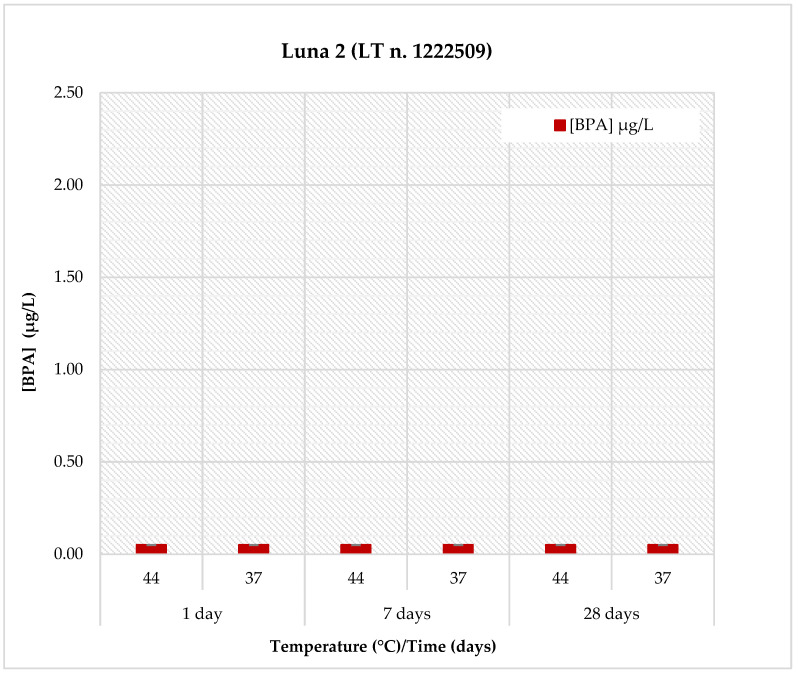
Comparison of BPA release in *Luna 2* material at pH = 6.8, two temperatures (44 °C and 37 °C) and at three different observation times (1, 7 and 28 days).

The BPA release profiles for all materials were evaluated in artificial saliva (pH: 6.8) under two temperature conditions (37 °C and 44 °C) and at three different time points (1, 7, and 28 days). The results are reported in Table 5 and illustrated in Figure 1. Detailed data for each individual material are shown in Figure 2, Figure 3, Figure 4 and Figure 5.

Figure 1 illustrates the overall BPA release patterns observed for the tested materials under the different temperature and time conditions. The highest BPA concentrations were generally observed at the elevated temperature (44 °C), with peak release occurring after 1 day of immersion in artificial saliva (pH 6.8). Notably, *Estelite Sigma Quick* exhibited the highest BPA release, reaching a peak concentration of 1.83 ± 0.22 µg/L after 1 day at 44 °C. *Clearfil Majesty ES-2* followed, showing the second-highest BPA release under the same conditions (1.41 ± 0.17 µg/L). In contrast, *Omnichroma Flow* released markedly lower amounts of BPA, with a maximum concentration of 0.58 ± 0.07 µg/L. Conversely, BPA concentrations for *Luna 2* remained consistently below the limit of quantification (<LOQ) under all tested conditions.

Figure 2 illustrates the BPA concentrations measured for *Omnichroma Flow*. In artificial saliva at physiological pH (pH 6.8), the highest BPA concentrations were observed at 44 °C, with peak values detected after 1 day of immersion (0.580 ± 0.070 µg/L). At the same temperature, lower concentrations were measured after 7 days (0.290 ± 0.035 µg/L), followed by a further decrease after 28 days (0.110 ± 0.013 µg/L). At 37 °C, BPA release followed a similar decreasing trend over time (1 day: 0.250 ± 0.030 µg/L; 7 days: 0.170 ± 0.020 µg/L; 28 days: <LOQ). These results indicate a temperature- and time-dependent BPA release pattern *for Omnichroma Flow*, with higher concentrations observed at elevated temperature and shorter immersion times.

The results for *Estelite Sigma Quick* are presented in Figure 3. In artificial saliva at physiological pH, the highest BPA release was observed after 1 day of immersion at 44 °C, reaching a maximum concentration of 1.83 ± 0.22 µg/L. A slightly lower concentration was detected at the same time point at 37 °C (1.39 ± 0.17 µg/L). BPA release decreased over time at both temperatures, with concentrations of 1.30 ± 0.16 µg/L (44 °C) and 1.20 ± 0.14 µg/L (37 °C) after 7 days and further declining to 0.92 ± 0.11 µg/L (44 °C) and 0.75 ± 0.09 µg/L (37 °C) after 28 days.

The results for *Clearfil Majesty ES-2* are presented in Figure 4. In artificial saliva, the highest BPA release was observed after 1 day of immersion at 44 °C, reaching a maximum concentration of 1.41 ± 0.17 µg/L. A lower concentration was detected at the same time point at 37 °C (0.87 ± 0.10 µg/L). BPA concentrations progressively decreased over time at both temperatures, measuring 0.75 ± 0.09 µg/L at 7 days and 0.40 ± 0.05 µg/L at 28 days at 44 °C, and 0.48 ± 0.058 µg/L at 7 days and 0.24 ± 0.03 µg/L at 28 days at 37 °C.

The results achieved for Luna 2 are presented in Figure 5, showing that only negligible BPA releasing was observed in artificial saliva, with concentrations remaining below 0.10 µg/L (<LOQ) under all tested conditions, including both temperatures and all time points.

The results provide a detailed assessment of BPA release from the tested resin-based dental materials in artificial saliva at physiological pH, showing clear patterns related to temperature, exposure duration, and material type. For all materials in which BPA release was detected, the highest concentrations were observed after 1 day of immersion at 44 °C. Distinct material-dependent differences were observed, with *Estelite Sigma Quick* showing the highest BPA release, followed by *Clearfil Majesty ES-2* and *Omnichroma Flow*, while *Luna 2* showed no quantifiable BPA release.

### 3.2. Results of Statistical Analysis

The statistical analysis demonstrated that BPA release from the tested dental restorative materials was significantly influenced by the composite material itself (*p* < 0.001), the temperature of the immersion medium (*p* < 0.001), and the exposure duration (*p* < 0.001).

Post hoc pairwise comparisons with Bonferroni correction revealed statistically significant differences between all tested materials (*p* < 0.001), except for the difference between *Omnichroma Flow* and *Clearfil Majesty ES-2*, which approached significance (*p* = 0.055). *Estelite Sigma Quick* showed the highest BPA release, with a mean concentration of 1.232 ± 0.063 µg/L, followed by *Clearfil Majesty ES-2* (0.688 ± 0.072 µg/L), *Omnichroma Flow* (0.243 ± 0.032 µg/L). BPA concentrations for *Luna 2* remained consistently below the LOQ and were therefore excluded from quantitative post hoc comparisons.

BPA concentrations were significantly higher at 44 °C compared to 37 °C (*p* < 0.001). BPA release peaked at day 1, then significantly decreased after 7 days (*p* < 0.001) and further after 28 days (*p* < 0.001). A significant interaction was observed between temperature and time (*p* < 0.001), indicating that the release pattern over time differed between the two tested temperatures. In contrast, the material × temperature interaction did not reach statistical significance (*p* = 0.505), suggesting that the temperature effect was similar across the different composite materials. The material × time interaction approached significance (*p* = 0.053), while the three-way material × temperature × time interaction was not significant (*p* = 0.336).

The Shapiro–Wilk test indicated that the distribution of BPA release values did not significantly deviate from normality when each material was considered separately (*p* > 0.20 for all materials).

## 4. Discussion

The results of the present study indicate that the organic chemical composition of the restorative material represents the primary determinant of BPA release, confirming that resin-based dental composites do not exhibit uniform chemical stability under different temperature and exposure conditions. In this sense, the null hypothesis was fully rejected. Quantitatively, *Estelite Sigma Quick* showed the highest BPA release, with peak concentrations of approximately 1.8 µg/L after 1 day at 44 °C. *Clearfil Majesty ES-2* also exhibited significant release levels, with peak values around 1.4 µg/L under the same conditions, whereas *Omnichroma Flow* released lower amounts, remaining below 0.6 µg/L even under thermal stress. In contrast, BPA concentrations for *Luna 2* were consistently below the limit of quantification (LOQ) at all time points and temperatures. These inter-material differences remained statistically robust after conservative post hoc correction. The borderline difference observed between *Clearfil Majesty ES-2* and *Omnichroma Flow* (*p* = 0.055) indicates that, although consistent numerical differences were present (Figure 1, Figure 2 and Figure 3), the available data do not provide sufficient statistical evidence to conclude that their BPA release profiles differ.

Concerns regarding the biocompatibility and long-term safety of resin-based dental restorative materials have increased alongside their widespread use in contemporary restorative dentistry [55]. Once placed in the oral cavity, these polymer-based materials are subjected to thermal, mechanical, chemical, and biological challenges that may promote material degradation and the release of residual monomers and degradation by-products [56,57,58]. A well-recognized limitation of methacrylate-based systems is incomplete monomer conversion during photo-polymerization, with reported degrees of conversion ranging from 50% to 80% [59], resulting in the potential release of low-molecular-weight compounds over time. Although typically present at low concentrations, the biological activity of these substances has warranted continued investigation into the biocompatibility of resin-based materials [60].

Within this context, BPA has received particular attention due to its classification as an endocrine-disrupting chemical and its documented biological activity at very low exposure levels [61,62]. BPA release from resin-based dental filling materials has been reported in laboratory studies; however, the literature is characterized by substantial heterogeneity in reported concentrations [21,22,23,24,27,30,42]. This variability largely reflects differences in extraction methods and detection techniques, making direct comparison across studies challenging. The present investigation addresses these limitations by employing a sensitive LC–MS/MS method and a standardized experimental design, thereby enabling a robust comparative evaluation of BPA release under controlled and clinically relevant conditions.

The mixed-effects statistical analysis demonstrated in this study that BPA release was significantly influenced by polymer composite material, temperature, and exposure duration (*p* < 0.001 for all main effects).

From a mechanistic perspective, the results above plotted and mentioned are consistent with differences in resin matrix chemistry. Materials containing bisphenol-derived monomers such as Bis-GMA are more likely to release BPA either as a residual contaminant from monomer synthesis or as a degradation product of ester bonds within the polymer network [63]. Conversely, Luna 2 is formulated primarily with urethane dimethacrylate (UDMA) and other monomers that are not structurally derived from bisphenol A. The absence of detectable BPA release from this material throughout the experimental period (<LOQ at all time points and temperatures) supports the interpretation that resin matrix chemistry represents a key determinant of BPA elution from polymer-based dental restorative materials. Composites formulated without bisphenol-derived monomers appear less likely to generate BPA as either residual impurity or degradation product under the tested conditions [64]. While filler loading, degree of conversion, and resin hydrophilicity may influence diffusion and degradation kinetics, the present results indicate that the presence or absence of bisphenol-related monomers represents the principal driver of BPA release under the tested conditions.

Temperature was identified as a strong and independent modulator of BPA release, with significantly higher concentrations detected at 44 °C compared to 37 °C across all materials (*p* < 0.001). Depending on the material and time point, BPA release at elevated temperature was consistently higher than that observed at 37 °C, with increases ranging from moderate to more pronounced depending on the material and time point. Notably, the absence of a significant material × temperature interaction (*p* = 0.505) indicates that the effect of temperature was broadly proportional among the tested composites. This finding suggests that thermal elevation acts as a general accelerator of diffusion and degradation processes within methacrylate-based resin matrices rather than selectively affecting specific formulations. Although 44 °C does not represent a sustained intraoral temperature, it was intentionally included as a stress condition to simulate transient thermal elevations associated with hot beverage consumption or febrile states [53,54]. Accordingly, the observed increase in BPA release at this temperature should be interpreted as a worst-case scenario rather than as representative of steady-state clinical conditions.

The temporal analysis (Figure 1) revealed a characteristic release pattern, with BPA concentrations peaking at day 1 and progressively decreasing at 7 and 28 days (*p* < 0.001 for time effect). Across bisphenol-A containing materials, a marked reduction in BPA release was observed over time, with decreases exceeding 50% by day 28 depending on material and temperature. This behavior is consistent with an initial burst release of surface-associated or loosely bound BPA, followed by depletion of readily releasable fractions. The significant temperature × time interaction (*p* < 0.001) further indicates that thermal stress modifies not only the magnitude but also the kinetics of BPA release, suggesting accelerated early elution and altered degradation dynamics at elevated temperature. In contrast, the absence of a significant three-way interaction among material, temperature, and time (*p* = 0.336) indicates that no resin-based composite exhibited a disproportionate or anomalous release behavior under combined experimental conditions. Although the present experimental design involved partial renewal of the artificial saliva medium at each sampling interval, which allowed interval-based measurement rather than direct cumulative quantification, the overall trend observed across the 28-day observation period indicates a progressive reduction in BPA release over time. This pattern suggests that the majority of BPA release occurs during the early phase after immersion, followed by a gradual depletion of the readily releasable fraction. Considering the exposed surface area of the specimens (approximately 95 mm^2^), the measured BPA concentrations can also be interpreted in relation to the specimen surface. The use of standardized specimen dimensions ensured comparable surface exposure among materials, allowing a consistent comparison of release behavior under the tested conditions. Because all specimens were prepared with identical dimensions and therefore identical exposed surface areas, the comparison of BPA concentrations among materials reflects differences in release behavior rather than differences in surface exposure.

These findings are consistent with previously published investigations evaluating the release of bisphenol-related compounds from resin-based dental materials and orthodontic adhesive systems. For instance, Małkiewicz et al. reported detectable release of BPA and related derivatives from several orthodontic adhesive systems, with the highest concentrations occurring during the early stages of incubation followed by a progressive decrease over time [65]. This temporal behavior closely resembles the release pattern observed in the present study, where BPA concentrations were highest after the first day of immersion and declined thereafter. Similarly, Lopes-Rocha et al. evaluated BPA and Bis-GMA release from contemporary dental composites using LC–MS/MS analysis and highlighted that the detection and magnitude of bisphenol-related compounds strongly depend on resin matrix composition and analytical sensitivity [30]. Differences in absolute BPA concentrations reported across studies may be attributed to variations in material formulation, degree of monomer conversion, extraction media, and experimental conditions. Taken together, these findings support the interpretation that BPA release from polymer-based dental materials is highly material-dependent and influenced by both resin chemistry and environmental factors [66].

When contextualized within limited available in vivo evidence, the BPA concentrations detected in this study are of the same order of magnitude as those reported in saliva following resin-based restorative procedures, warranting consideration within a cumulative exposure framework [25,67]. Nevertheless, the toxicological relevance of BPA is increasingly interpreted within a cumulative exposure framework. The recent revision of the tolerable daily intake by the European Food Safety Authority to 0.2 ng/kg body weight/day highlights that even small and transient exposure sources may contribute meaningfully to overall BPA burden [35]. In this context, dental polymer restorative materials represent a localized but persistent source of potential exposure, particularly given their long-term presence in the oral cavity and the frequent need for multiple restorations. Although the absolute BPA concentrations detected in the present study were low, their potential contribution should be interpreted within the broader context of cumulative human exposure. Dietary intake is considered the primary source of BPA exposure in the general population [38]; however, additional contributions from environmental sources and consumer products, including grinding and wear of dental restorative materials, may incrementally increase total body burden [39]. In this respect, even localized and transient release from dental materials may be relevant when considered alongside other continuous exposure pathways. Importantly, several in vitro studies have reported biological activity of BPA at very low concentrations, including effects on endocrine signaling, oxidative stress, and inflammatory pathways in cell-based models, suggesting that toxicological relevance cannot be evaluated solely based on acute toxicity thresholds [68]. However, the present study did not evaluate biological effects, and therefore these observations should be interpreted only as contextual toxicological evidence reported in the literature. Nevertheless, the present investigation is inherently limited by its in vitro design, and any extrapolation to in vivo exposure or clinical risk must be made with caution. The findings should therefore be interpreted as comparative evidence of material-dependent BPA release under controlled conditions, rather than as an evaluation of the impact on human health.

An important unresolved issue concerns the precise molecular origin of BPA detected in dental resin-based composite materials, including those marketed as “BPA-free” [69,70]. Although Bis-GMA was historically synthesized from BPA, most contemporary formulations no longer use BPA as a direct starting compound [45,70]. Therefore, the measurable BPA concentrations detected in this study may arise from alternative degradation pathways involving other aromatic dimethacrylates, such as Bis-EMA or Bis-DMA, or from trace contamination introduced during manufacturing, packaging, or storage. While the present findings confirm BPA release under specific experimental conditions, they do not allow definitive attribution of its molecular source, underscoring the need for further targeted investigations. From a clinical perspective, these results suggest that, whenever feasible, restorative dental polymer materials demonstrating consistently low or negligible BPA release under stress conditions may be preferred, particularly in patients with extensive restorative needs, where the level of dental wear increases, in bruxist patients, or in vulnerable populations. When bisphenol-containing materials are indicated for esthetic or mechanical reasons, the previous literature suggests that exposure-minimizing clinical protocols, such as rubber dam isolation, immediate finishing and polishing, removal of the oxygen-inhibited layer, and thorough rinsing, may help reduce early BPA release [71,72,73], although these procedures were not directly evaluated in the present study.

### Limitations and Future Directions

Despite the use of a sensitive analytical methodology and controlled experimental conditions, the present study has several limitations that should be acknowledged. First, the in vitro design cannot fully reproduce the complexity of the oral environment, including salivary flow, enzymatic activity, biofilm formation, mechanical loading and wear, and dynamic pH fluctuations. Furthermore, the polishing procedure used in the present study was intended to standardize surface conditions across all specimens and therefore does not fully reproduce the multi-step finishing and polishing procedures typically performed in clinical practice. Future studies should evaluate how different clinical finishing protocols may influence the release of BPA from resin-based composites.

Second, the observation period was limited to 28 days, which provides insight into the time-dependent in vitro release behavior of BPA under the experimental conditions adopted in the present study, but does not allow definitive prediction of BPA release over the prolonged periods during which dental restorations typically remain in the oral cavity contributing to mastication and wear. Indeed, future investigations should evaluate the effect of mechanical surface challenges on BPA release from resin-based composites. Simulated toothbrushing or controlled surface abrasion applied after extended immersion periods may help clarify whether removal of the superficial resin layer, following apparent depletion of readily releasable BPA fractions, could induce secondary BPA release from deeper regions of the material. Such protocols would better reflect clinically relevant aging processes associated with mastication, oral hygiene practices, and progressive surface wear over time.

Third, although the sample size was sufficient to detect large effects, smaller differences between materials may have gone undetected and warrant investigation in larger cohorts.

Fourth, the absence of mechanical loading and thermocycling protocols represents another limitation of the present study. Although the elevated temperature of 44 °C was included to simulate transient thermal stress conditions, such temperatures are not continuously sustained under in vivo conditions. However, the primary aim of this investigation was to characterize BPA release under controlled chemical and thermal conditions, allowing direct comparison of material-dependent release behavior without the confounding effects of fatigue-induced surface damage. Future studies should therefore integrate dynamic thermal cycling and mechanical fatigue protocols, as recommended for aging procedures in dental materials testing by ISO 11405 and ISO 10477 (typically 5–55 °C, 500–10,000 cycles), to further contextualize these findings within clinically relevant aging scenarios [74,75].

An additional limitation concerns the potential contribution of external contamination sources, such as plastic delivery capsules, which were not specifically controlled for in the present study. Inclusion of capsule-only controls and expanded analytical targeting of non-Bis-GMA degradation products should be prioritized in future research. Finally, long-term in vivo studies assessing cumulative BPA exposure in patients with multiple restorations, together with the development of truly bisphenol-free restorative systems that maintain high mechanical performance, remain essential objectives for future investigation.

## 5. Conclusions

Despite the limitations of this in vitro study, BPA release from resin-based dental composites was shown to be material-dependent and influenced by temperature and exposure duration. Detectable BPA release was primarily observed during early immersion phases and under elevated temperature in materials containing bisphenol-derived monomers, whereas the UDMA-based composite (*Luna 2*) consistently exhibited BPA levels below the LOQ. Although the absolute concentrations detected were low, the results highlight the relevance of resin matrix composition in governing elution behavior and support the need for continued evaluation of BPA release within a cumulative exposure perspective.

Overall, the results underline the role of resin matrix formulation in influencing BPA release while confirming that contemporary resin-based composites, when appropriately selected and handled, remain suitable materials for restorative dentistry. Ongoing material optimization, together with prudent clinical protocols and independent analytical assessment, may further contribute to minimizing unnecessary exposure without compromising the established clinical performance of these restorative systems.

## Figures and Tables

**Table 1:** Composite material specifications.

Material	Manufacturer	Type	Curing Mechanism	Composition
**Omnichroma Flow** (LT n. 041M45)	Tokuyama Dental (Tokyo, Japan)	Resin composite	Light curing	71% by weight (57% by volume) of spherical silica-zirconia filler and composite filler. 1,6-bis (methacryl ethyloxycarbonylamino) trimethyl hexane (UDMA), 1,9-nonamethylene glycol dimethacrylate, Mequinol, Dibutyl hydroxyl toluene and UV absorber.
**Estelite sigma quick** (LT n. 350M45)	Tokuyama Dental (Tokyo, Japan)	Resin composite	Light curing	Bis-GMA, TEGDMA, Composite filler, Silica-zirconia filler, Photo initiator
**Clearfil Majesty ES-2** (LT n. 5F0093)	Kuraray Co., Ltd. (Osaka, Japan)	Resin composite	Light curing	Bis-GMA, Hydrophobic aliphatic dimethacrylate, Silanated barium glass filler, Organic filler/78 wt% Silanated barium glass filler: 0.7 μm Organic filler: <100 μm
**Luna 2** (LT n. 1222509)	SDI (Victoria, Australia)	Resin composite	Light curing	UDMA, TEGDMA, TCD-DMA, Sr glass, SiO_2_ (hydrophobic fumed silica), YbF_3_, Initiators, Stabilizers, Pigments

UDMA: urethane dimethacrylate; Bis-GMA: bisphenol-glycidyl methacrylate; TEGDMA: Triethylene glycol dimethacrylate; TCD-DMA: Tricyclodecane-Dimethanol Diacrylate; YbF_3_: ytterbium trifluoride; LT n.: lot number.

**Table 2:** Fusayama Meyer’s artificial saliva solution components.

Component	Concentration: g/L
Potassium chloride (KCl)	0.4
Sodium chloride (NaCl)	0.4
Calcium chloride dihydrate (CaCl_2_·2H_2_O)	0.906
Monosodium phosphate dihydrate (NaH_2_PO_4_·2H_2_O)	0.69
Sodium sulfide nonahydrate (Na_2_S·9H_2_O)	0.005
Urea	1.0

**Table 3:** Gradient chromatographic elution optimized to separate BPA.

Time	Ultrapure Water with 0.01% Acetic Acid	MeOH Water with 0.01% Acetic Acid
0.0	60.0	40.0
0.5	60.0	40.0
3.0	5.0	95.0
4.0	5.0	95.0

**Table 4:** UHPLC–MS/MS quantifier and qualifier transitions employed for the determination of BPA.

Ion	Transition (Q1 → Q3)	Collision Energy (eV)	Fragmentor (V)	Function
BPA-Q	227.2 → 133.0	−20	162	Quantifier
BPA-q	227.2 → 211.8	−28	162	Qualifier

**Table 5:** Mean concentrations (µg/L) ± standard deviation (SD) for the different materials measured in artificial saliva (pH = 6.8), at two temperatures (37 °C and 44 °C) and three observation times (1, 7 and 28 days).

Parameters			Materials			
pH	Time	T (°C)	Omnichroma Flow (LT n. 041M45)	Estelite Sigma Quick (LT n. 350M45)	Clearfil Majesty ES-2 (LT n. 5F0093)	Luna 2 (LT n. 1222509)
6.8	1 day	44	0.580 ± 0.070	1.83 ± 0.22	1.41 ± 0.17	<LOQ
		37	0.250 ± 0.030	1.39 ± 0.17	0.87 ± 0.10	<LOQ
	7 days	44	0.290 ± 0.035	1.30 ± 0.16	0.75 ± 0.09	<LOQ
		37	0.170 ± 0.020	1.20 ± 0.14	0.48 ± 0.058	<LOQ
	28 days	44	0.110 ± 0.013	0.92 ± 0.11	0.40 ± 0.05	<LOQ
		37	<LOQ	0.75 ± 0.09	0.22 ± 0.03	<LOQ

## Data Availability

The original contributions presented in this study are included in the article. Further inquiries can be directed to the corresponding author.

## References

[1] Ilie N., Hickel R. (2011). Resin composite restorative materials. Aust. Dent. J..

[2] Ferracane J.L. (2011). Resin composite—State of the art. Dent. Mater..

[3] German M.J. (2022). Developments in resin-based composites. Br. Dent. J..

[4] Cramer N.B., Stansbury J.W., Bowman C.N. (2011). Recent advances and developments in composite dental restorative materials. J. Dent. Res..

[5] Fugolin A.P.P., Pfeifer C.S. (2017). New Resins for Dental Composites. J. Dent. Res..

[6] Szczesio-Wlodarczyk A., Sokolowski J., Kleczewska J., Bociong K. (2020). Ageing of Dental Composites Based on Methacrylate Resins—A Critical Review of the Causes and Method of Assessment. Polymers.

[7] Leung B.A., Joe W., Mofarah S.S., Sorrell C.C., Abbasi R., Azadeh M., Arsecularatne J.A., Koshy P. (2023). Unveiling the mechanisms behind surface degradation of dental resin composites in simulated oral environments. J. Mater. Chem. B..

[8] Meereis C.T.W., Münchow E.A., de Oliveira da Rosa W.L., da Silva A.F., Piva E. (2018). Polymerization shrinkage stress of resin-based dental materials: A systematic review and meta-analyses of composition strategies. J. Mech. Behav. Biomed. Mater..

[9] Putzeys E., De Nys S., Cokic S.M., Duca R.C., Vanoirbeek J., Godderis L., Meerbeek B.V., Van Landuyt K.L. (2019). Long-term elution of monomers from resin-based dental composites. Dent. Mater..

[10] Guo X., Yu Y., Gao S., Zhang Z., Zhao H. (2022). Biodegradation of Dental Resin-Based Composite—A Potential Factor Affecting the Bonding Effect: A Narrative Review. Biomedicines.

[11] Randolph L.D., Palin W.M., Leloup G., Leprince J.G. (2016). Filler characteristics of modern dental resin composites and their influence on physico-mechanical properties. Dent. Mater..

[12] Szczesio-Wlodarczyk A., Domarecka M., Kopacz K., Sokolowski J., Bociong K. (2021). An Evaluation of the Properties of Urethane Dimethacrylate-Based Dental Resins. Materials.

[13] Barszczewska-Rybarek I.M., Chrószcz M.W., Chladek G. (2020). Novel Urethane-Dimethacrylate Monomers and Compositions for Use as Matrices in Dental Restorative Materials. Int. J. Mol. Sci..

[14] Berghaus E., Klocke T., Maletz R., Petersen S. (2023). Degree of conversion and residual monomer elution of 3D-printed, milled and self-cured resin-based composite materials for temporary dental crowns and bridges. J. Mater. Sci. Mater. Med..

[15] Grutle L.A., Holm H.V., Kopperud H.B.M., Uhlig S. (2024). Validation of a human saliva model for the determination of leachable monomers and other chemicals from dental materials. J. Chromatogr. B Anal. Technol. Biomed. Life Sci..

[16] Janani K., Teja K.V., Sandhya R., Alam M.K., Al-Qaisi R.K., Shrivastava D., Alnusayri M.O., Alkhalaf Z.A., Sghaireen M.G., Srivastava K.C. (2021). Monomer Elution from Three Resin Composites at Two Different Time Interval Using High Performance Liquid Chromatography—An In-Vitro Study. Polymers.

[17] Ilie N. (2022). Degradation of Dental Methacrylate-Based Composites in Simulated Clinical Immersion Media. J. Funct. Biomater..

[18] Lopes-Rocha L., Ribeiro-Gonçalves L., Henriques B., Özcan M., Tiritan M.E., Souza J.C.M. (2021). An integrative review on the toxicity of Bisphenol A (BPA) released from resin composites used in dentistry. J. Biomed. Mater. Res. B Appl. Biomater..

[19] Hahladakis J.N., Iacovidou E., Gerassimidou S. (2023). An overview of the occurrence, fate, and human risks of the bisphenol—A present in plastic materials, components, and products. Integr. Environ. Assess. Manag..

[20] Abraham A., Chakraborty P. (2020). A review on sources and health impacts of bisphenol A. Rev. Environ. Health.

[21] Tichy A., Simkova M., Vrbova R., Roubickova A., Duskova M., Bradna P. (2021). Bisphenol A Release from Dental Composites and Resin-Modified Glass Ionomers under Two Polymerization Conditions. Polymers.

[22] De Nys S., Duca R.C., Vervliet P., Covaci A., Boonen I., Elskens M., Vanoirbeek J., Godderis L., Van Meerbeek B., Van Landuyt K.L. (2021). Bisphenol A as degradation product of monomers used in resin-based dental materials. Dent. Mater..

[23] De Nys S., Duca R.C., Vervliet P., Covaci A., Boonen I., Elskens M., Vanoirbeek J., Godderis L., Van Meerbeek B., Van Landuyt K.L. (2022). Bisphenol A release from short-term degraded resin-based dental materials. J. Dent..

[24] De Nys S., Turkalj M., Duca R.C., Covaci A., Elskens M., Godderis L., Vanoirbeek J., Van Meerbeek B., Van Landuyt K.L. (2024). Level of BPA contamination in resin composites determines BPA release. Dent. Mater..

[25] Berge T.L.L., Lygre G.B., Lie S.A., Lindh C.H., Björkman L. (2019). Bisphenol A in human saliva and urine before and after treatment with dental polymer-based restorative materials. Eur. J. Oral Sci..

[26] Vervliet P., De Nys S., Duca R.C., Boonen I., Godderis L., Elskens M., Van Landuyt K.L., Covaci A. (2023). Degradation products of resin-based materials detected in saliva in vivo. Clin. Oral Investig..

[27] De Nys S., Putzeys E., Duca R.C., Vervliet P., Covaci A., Boonen I., Elskens M., Vanoirbeek J., Godderis L., Van Meerbeek B. (2021). Long-term elution of bisphenol A from dental composites. Dent. Mater..

[28] Tichy A., Srolerova T., Schwendicke F. (2025). Release of Bisphenol A from Dental Materials: Risks and Future Perspectives. J. Dent. Res..

[29] De Angelis F., Sarteur N., Buonvivere M., Vadini M., Šteffl M., D’Arcangelo C. (2022). Meta-analytical analysis on components released from resin-based dental materials. Clin. Oral Investig..

[30] Lopes-Rocha L., Gonçalves V.M.F., Cunha S.C., Fernandes J.O., Pinho T., Tiritan M.E. (2023). Evaluation of BPA and Bis-GMA Release from Recent Dental Composite Materials by LC-MS/MS. Separations.

[31] Stanojević M., Sollner Dolenc M. (2025). Mechanisms of bisphenol A and its analogs as endocrine disruptors via nuclear receptors and related signaling pathways. Arch. Toxicol..

[32] Park C., Song H., Choi J., Sim S., Kojima H., Park J., Iida M., Lee Y. (2020). The mixture effects of bisphenol derivatives on estrogen receptor and androgen receptor. Environ. Pollut..

[33] Rubin B.S. (2011). Bisphenol A: An endocrine disruptor with widespread exposure and multiple effects. J. Steroid Biochem. Mol. Biol..

[34] Keteci F., Bakan B. (2025). Comparative Analysis of Bisphenol A and Its Derivatives (BPF and BPS) on Oxidative Injury and Apoptosis in Dermal Fibroblasts. J. Appl. Toxicol..

[35] Lambré C., Barat Baviera J.M., Bolognesi C., Chesson A., Cocconcelli P.S., Crebelli R., Gott D.M., Grob K., Lampi E., EFSA Panel on Food Contact Materials, Enzymes and Processing Aids (CEP) (2023). Re-evaluation of the risks to public health related to the presence of bisphenol A (BPA) in foodstuffs. EFSA J..

[36] Rochester J.R. (2013). Bisphenol A and human health: A review of the literature. Reprod. Toxicol..

[37] Berger K., Eskenazi B., Kogut K., Parra K., Lustig R.H., Greenspan L.C., Holland N., Calafat A.M., Ye X., Harley K.G. (2018). Association of Prenatal Urinary Concentrations of Phthalates and Bisphenol A and Pubertal Timing in Boys and Girls. Environ. Health Perspect..

[38] Manzoor M.F., Tariq T., Fatima B., Sahar A., Tariq F., Munir S., Khan S., Nawaz Ranjha M.M.A., Sameen A., Zeng X.A. (2022). An insight into bisphenol A, food exposure and its adverse effects on health: A review. Front. Nutr..

[39] Bobadilla-Lozoya K., Morales-Montor J., Mejía-Salgado K.G., Nava-Castro K.E. (2026). The emerging pollutants polycyclic aromatic compounds (PAH’S) bisphenol a (BPA), and phthalates impair immune system function: Effects on human macrophages. Toxicol. Vitr..

[40] Almeida S., Raposo A., Almeida-González M., Carrascosa C. (2018). Bisphenol A: Food Exposure and Impact on Human Health. Compr. Rev. Food Sci. Food Saf..

[41] Bello-Cortes I.H., García-García J.A., Gutiérrez-Aguilar M., Araiza-Olivera D., Sánchez-Pérez C., García-Cerón G., Morán-Ramos S., Tovar H., Bonilla-Brunner A., García-Arrazola R. (2025). Prenatal exposure to Bisphenol-A as a risk factor for infant neurodevelopment. Front. Endocrinol..

[42] Hampe T., Wiessner A., Frauendorf H., Alhussein M., Karlovsky P., Bürgers R., Krohn S. (2022). Monomer Release from Dental Resins: The Current Status on Study Setup, Detection and Quantification for In Vitro Testing. Polymers.

[43] Ahmad H., Mills D., Davis G., Baysan A. (2025). Bioactive resin composite with the potential of ion exchange following selective carious lesion removal—A laboratory-based study. J. Dent..

[44] Mahmoudi Meimand N., Tsoi J.K.H., Burrow M.F., He J., Cho K. (2024). A comparative study on the mechanical and antibacterial properties of BPA-free dental resin composites. Dent. Mater..

[45] Sun Y., Zhou Z., Jiang H., Duan Y., Li J., Liu X., Hong L., Zhao C. (2022). Preparation and evaluation of novel bio-based Bis-GMA-free dental composites with low estrogenic activity. Dent. Mater..

[46] Aliberti A., Garcia-Godoy F., Borges A.L.S., Tribst J.P.M., Gasparro R., Mariniello M., Ausiello P. (2025). Calcium, phosphate and fluoride ionic release from dental restorative materials for elderly population: An in vitro analysis. Front. Oral Health.

[47] Leung V.W., Darvell B.W. (1997). Artificial salivas for in vitro studies of dental materials. J. Dent..

[48] Nigam A.G., Jaiswal J., Murthy R., Pandey R. (2009). Estimation of fluoride release from various dental materials in different media—An in vitro study. Int. J. Clin. Pediatr. Dent..

[49] Koda T., Tsuchiya H., Yamauchi M., Ohtani S., Takagi N., Kawano J. (1990). Leachability of denture-base acrylic resins in artificial saliva. Dent. Mater..

[50] Patel B., Duran-Martinez A.C., Gurman P., Auciello O., Barao V., Campbell S., Sukotjo C., Mathew T.M. (2017). Ultrananocrystalline diamond coatings for the dental implant: Electrochemical nature. Surf. Innov..

[51] Aliberti A., Di Duca F., Triassi M., Montuori P., Scippa S., Piscopo M., Ausiello P. (2025). The Effect of Different pH and Temperature Values on Ca^2+^, F^−^, PO_4_^3−^, OH^−^, Si, and Sr^2+^ Release from Different Bioactive Restorative Dental Materials: An In Vitro Study. Polymers.

[52] Aliberti A., Gasparro R., Triassi M., Piscopo M., Ausiello P., Tribst J.P.M. (2025). Fluoride Release from Pediatric Dental Restorative Materials: A Laboratory Investigation. Dent. J..

[53] Airoldi G., Riva G., Vanelli M., Filippi V., Garattini G. (1997). Oral environment temperature changes induced by cold/hot liquid intake. Am. J. Orthod. Dentofac. Orthop..

[54] Cramer M.N., Gagnon D., Laitano O., Crandall C.G. (2022). Human temperature regulation under heat stress in health, disease, and injury. Physiol. Rev..

[55] Sales-Junior R.A., de Bessa M.S., Oliveira F.J.D., Barbosa B.F.S., Santos K.S., Owen M., Feitosa V.P., Borges B.C.D. (2025). Multifaceted characterization of antibacterial resin composites: A scoping review on efficacy, properties, and in vivo performance. Jpn. Dent. Sci. Rev..

[56] Basharat K.A., Alam S.A., Hussain A., Farooq A., Bokhari S.E., Malik A.A. (2025). Effects of ph and Temperature on Dental Materials in Saliva and Oral Fluids. Int. J. Pharm. Res. Technol..

[57] di Lauro A.E., Ciaramella S., Tribst J.P.M., Aliberti A., Ausiello P. (2024). Comparison of Bulk Polymeric Resin Composite and Hybrid Glass Ionomer Cement in Adhesive Class I Dental Restorations: A 3D Finite Element Analysis. Polymers.

[58] Grassi E.D.A., de Andrade G.S., de Carvalho A.B.G., Gasparro R., Mariniello M., Aliberti A., Ausiello P., Borges A.L.S. (2025). Evaluation of Internal and Marginal Shrinkage Stress in Adhesive Class III Cavities Restored with Different Resin Composite Combinations—A 3D-FEA Study. Dent. J..

[59] Lehmann A., Nijakowski K., Drożdżyńska A., Przybylak M., Woś P., Surdacka A. (2022). Influence of the Polymerization Modes on the Methacrylic Acid Release from Dental Light-Cured Materials—In Vitro Study. Materials.

[60] Ausiello P., Dal Piva A.M.d.O., Borges A.L.S., Lanzotti A., Zamparini F., Epifania E., Mendes Tribst J.P. (2021). Effect of Shrinking and No Shrinking Dentine and Enamel Replacing Materials in Posterior Restoration: A 3D-FEA Study. Appl. Sci..

[61] Hafezi S.A., Abdel-Rahman W.M. (2019). The Endocrine Disruptor Bisphenol A (BPA) Exerts a Wide Range of Effects in Carcinogenesis and Response to Therapy. Curr. Mol. Pharmacol..

[62] Emfietzoglou R., Spyrou N., Mantzoros C.S., Dalamaga M. (2019). Could the endocrine disruptor bisphenol-A be implicated in the pathogenesis of oral and oropharyngeal cancer? Metabolic considerations and future directions. Metabolism.

[63] Rogocka M., Lewusz-Butkiewicz K., Marek E., Mazurek-Mochol M., Łagocka R. (2023). The toxicity of Bis-GMA, a basic monomer of the dental composite’s organic matrix—A narrative review. Pomeranian J. Life Sci..

[64] Hampe T., Liersch J., Wiechens B., Wassmann T., Schubert A., Alhussein M., Bürgers R., Krohn S. (2022). A Pilot Study on Monomer and Bisphenol A (BPA) Release from UDMA-Based and Conventional Indirect Veneering Composites. Polymers.

[65] Małkiewicz K., Turło J., Marciniuk-Kluska A., Grzech-Leśniak K., Gąsior M., Kluska M. (2015). Release of bisphenol A and its derivatives from orthodontic adhesive systems available on the European market as a potential health risk factor. Ann. Agric. Environ. Med..

[66] Löfroth M., Ghasemimehr M., Falk A., Vult von Steyern P. (2019). Bisphenol A in dental materials—Existence, leakage and biological effects. Heliyon.

[67] Kingman A., Hyman J., Masten S.A., Jayaram B., Smith C., Eichmiller F., Arnold M.C., Wong P.A., Schaeffer J.M., Solanki S. (2012). Bisphenol A and other compounds in human saliva and urine associated with the placement of composite restorations. J. Am. Dent. Assoc..

[68] Gaivão I., Santos R.A., Morozova T.V., Tkach V.V. (2025). Biological and Behavioural Effects of Bisphenol A (BPA) Exposure: An In Vivo Study in Drosophila melanogaster. Appl. Sci..

[69] Deng H., Liu F., He J. (2024). The Effect of Inorganic Filler Content on the Properties of BPA-Free Bulk-Fill Dental Resin Composites. Materials.

[70] Tang C., Ahmed M.H., Yoshihara K., Peumans M., Van Meerbeek B. (2024). Multi-Parameter Characterization of HEMA/BPA-free 1- and 2-step Universal Adhesives Bonded to Dentin. J. Adhes. Dent..

[71] Luo S., Zhu W., Liu F., He J. (2016). Preparation of a Bis-GMA-Free Dental Resin System with Synthesized Fluorinated Dimethacrylate Monomers. Int. J. Mol. Sci..

[72] Paula A.B., Toste D., Marinho A., Amaro I., Marto C.-M., Coelho A., Marques-Ferreira M., Carrilho E. (2019). Once Resin Composites and Dental Sealants Release Bisphenol-A, How Might This Affect Our Clinical Management?—A Systematic Review. Int. J. Environ. Res. Public Health.

[73] Marzouk T., Sathyanarayana S., Kim A.S., Seminario A.L., McKinney C.M. (2019). A Systematic Review of Exposure to Bisphenol A from Dental Treatment. JDR Clin. Transl. Res..

[74] (2015). Dentistry—Testing of Adhesion to Tooth Structure.

[75] (2020). Dentistry—Polymer-Based Crown and Veneering Materials.

